# Esophageal perforation caused by external air-blast injury

**DOI:** 10.1186/1749-8090-5-130

**Published:** 2010-12-17

**Authors:** Jun-Neng Roan, Ming-Ho Wu

**Affiliations:** 1 Department of Surgery, Tainan Municipal Hospital, Tainan, Taiwan; 2 Department of Surgery, E-Da Hospital, Kaohsiung County, Taiwan

## Abstract

**Background:**

Esophageal perforation after external air-blast trauma is rarely presented in the emergency room. The diagnosis is often delayed more than 24 hours.

**Methods:**

We review the literature and report a case of esophageal perforation caused by external air-blast injury.

**Results:**

Including the present case, a total of 5 cases of esophageal perforation were caused by external air-blast injury in English literature. Of them, the common presentations were chest pain and dyspnea. The treatment methods varied with each case. One patient died before diagnosis of esophageal perforation and the others survived after proper surgical management.

**Conclusions:**

Early diagnosis and proper surgical management can reduce the morbidity and mortality of patients who suffered from esophageal perforation caused by external air-blast injury.

## Background

Esophageal perforation caused by air-blast injury is uncommon. An external air impact on the chest wall and upper abdomen, inducing rupture of the esophagus, is an even rare event. Only four cases of esophageal rupture caused by an external air-blast injury were found in a perusal of the English literature [1-4]. The objective of this article is to report our patient and a review of the literature to establish diagnostic and treatment strategies for esophageal perforation after an external air-blast injury.

## Case presentation

### Present Case

A 31-year-old man was struck on the right side of the face and left subcostal region at work when a nitrogen tank exploded four hours after he had eaten his lunch. He was knocked down to the ground and dazed, without loss of consciousness. He was immediately sent to the emergency department with a presentation of left chest pain and dyspnea. His vital signs were stable on arrival. An emergency left tube thoracostomy was performed, because his left-sided breath sounds had decreased, with a suspicion of pneumothorax. Ecchymosis and tenderness were detected on the left lower chest, without peritoneal signs. The patient was admitted for further observation and was allowed to intake thereafter. Esophageal rupture was not diagnosed until 84 hours after the injury when the tomato juice the patient had ingested was found in the chest bottle. Upon urgent left thoracotomy, a 4.5 cm laceration was found in the lower third of the esophagus, with severe inflammatory changes in the surrounding tissue. The pleural surface of the adjacent thoracic aorta was also torn. The esophagus was repaired with single-layer interrupted sutures of 3-0 Maxon (Davis & Geck, Wayne, NJ). Bile-containing fluid was predominantly expelled from the chest tube 48 hours after the primary repair. The patient underwent a subsequent transhiatal esophagectomy and reconstruction of the esophagus with ileocolon via the retrosternal route. With aggressive antibiotic treatment and total parenteral nutrition, his general condition was stabilized. He tolerated a full diet seven days postoperatively and was discharged 28 days after the injury. The patient was doing well during the follow-up period of more than five years.

### Results

Five cases including the present case of esophageal perforation caused by external air-blast injury were reviewed (Table 1). Of these five patients, one with cervical esophageal injury developed a tracheoesophageal fistula. Postoperative leakage occurred in two patients after primary repair of the thoracic esophagus. Among these five patients, one died before the diagnosis of esophageal perforation was made, and the others survived after proper surgical management.

**Table 1:** Esophageal Rupture Caused by External Air-Blast Injury

Author	Age	Interval to Diagnosis	Presentation	Injury of esophageal Site	Treatment	Complications	Outcome
Majeski [1]	15	48 hours	Chest pain, dyspnea	Lower	None		Died
Michel [2]	*	24 hours	*	Lower	Staged repair: Exclusion-diversion and subsequent esophageal replacement	None	Survived
Guth [3]	35	24 hours	Dyspnea	Middle	Primary repair with gastrostomy and feeding jejunostomy	Leakage and adult respiratory distress syndrome	Survived
Volk [4]	22	240 hours	Hemoptysis, subcutaneous emphysema, dysphagia	Cervical	Drainage and subsequent repair	Tracheoesophageal fistula	Survived
Roan**	31	84 hours	Chest pain	Lower	Primary repair and subsequent esophageal replacement	Leakage of primary repair	Survived

*Not reported, **the present case.

### Discussion

The incidence of esophageal perforation caused by blast trauma is estimated to be 0.004%-0.01% [2,3]. The most commonly reported mechanism of injury is high-pressure air directed into the esophagus via the mouth [3]. An external air-blast contusion on the anterior chest and abdomen that induces rupture of the esophagus, as showed in Table 1, is even rare [1-4]. The esophagus was predominately ruptured in the lower third (3/5 patients). External blast injury is caused by an impact on the anterior chest and upper abdomen that forms a shock wave in the body. The force is then predominantly conducted to the air-containing (hollow) organs, including the stomach [5]. Esophageal perforation can then occur from the sudden impact of the stomach gas.

The diagnosis of esophageal perforation is relatively difficult when the perforation site is located in the lower thoracic region. The most common symptoms are chest pain and dyspnea, which are not specific for esophageal rupture. Pneumomediastinum and pneumothorax are the most common findings in patients suffering from lower esophageal perforation [3]. An esophagogram should be considered for patients suffering from external air-blast injury who presented symptoms of chest pain, dyspnea or subcutaneous emphysema.

A diagnosis of thoracic esophageal perforation delayed for more than 24 hours could result in high morbidity and mortality rates. Surgical procedures include simple drainage, primary repair, esophageal exclusion with gastrostomy or jejunostomy, and esophagectomy followed by esophageal reconstruction. Primary repair is the most common procedure for blast-induced esophageal perforation. Guth et al. performed primary repair, with gastrostomy and feeding jejunostomy for early enteral nutrition support, when esophageal perforation was diagnosed within 24 hours [3]. Although the successful primary repair of an esophageal perforation has been reported even after the diagnosis had been delayed for more than 72 hours [6], leakage and mediastinal infection are common after the procedure. We performed a primary repair 84 hours after the injury, which was unsuccessful, so a subsequent transhiatal esophagectomy with a retrosternal ileocolon reconstruction was performed.

## Conclusions

Esophageal rupture should be suspected in patients suffering pneumothorax or pneumomediastinum after an external air-blast injury. Esophagogram followed by a high clinical suspicision after trauma is important for an early detection. Primary repair of the esophagus could be performed when the diagnosis was early. Esophageal exclusion or resection should be considered once the diagnosis has been delayed for more than 24 hours.

## Consent

Written informed consent was obtained from the patient for publication of this case report.

## Competing interests

The authors declare that they have no competing interests.

## Authors' contributions

JNR conceived of the study, gathered the data and wrote the manuscript. MHW participated in the design and coordination and overlooked the progress of the manuscript and advised on valuable amendments. Both authors read and approved the final manuscript.
